# *PRDX6AS1* gene polymorphisms and SLE susceptibility in Chinese populations

**DOI:** 10.3389/fimmu.2022.987385

**Published:** 2022-10-12

**Authors:** Xiao-Xue Zhang, Jun-Peng You, Xin-Ran Liu, Ya-Fei Zhao, Yan Cui, Zhan-Zheng Zhao, Yuan-Yuan Qi

**Affiliations:** ^1^Nephrology Hospital, The First Affiliated Hospital of Zhengzhou University, Zhengzhou University, Zhengzhou, China; ^2^Department of Nephrology, Wuhan Fourth Hospital, Puai Hospital, Tongji Medical College, Huazhong University of Science and Technology, Wuhan, China

**Keywords:** systemic lupus erythematosus, single nucleotide polymorphisms, *PRDX6-AS1*, lncRNA, rs844649

## Abstract

**Background:**

Systemic lupus erythematosus (SLE) is a complex, multisystem autoimmune disease that is characterized by the production of autoantibodies. Although accumulated evidence suggests that the dysregulation of long non-coding RNAs (lncRNAs) is involved in the pathogenesis of SLE, the genetic contributions of lncRNA coding genes to SLE susceptibility remain largely unknown. Here, we aimed to provide more evidence for the role of lncRNA coding genes to SLE susceptibility.

**Methods:**

The genetic association analysis was first adopted from the previous genome-wide association studies (GWAS) and was then validated in an independent cohort. *PRDX6-AS1* is located at chr1:173204199-173446294. It spans a region of approximately 240 kb, and 297 single nucleotide polymorphisms (SNPs) were covered by the previous GWAS. Differential expression at the mRNA level was analyzed based on the ArrayExpress Archive database.

**Results:**

A total of 33 SNPs were associated with SLE susceptibility, with a *P*<1.68×10^-4^. The strongest association signal was detected at rs844649 (*P*=2.12×10^-6^), according to the previous GWAS. Combining the results from the GWAS Chinese cohort and our replication cohort, we pursued a meta-analysis approach and found a pronounced genetic association between *PRDX6-AS1* rs844649 and SLE susceptibility (p_meta_=1.24×10^-13^, OR 1.50, 95% CI: 1.34–1.67). The mRNA expression of *PRDX6* was elevated in peripheral blood cells, peripheral blood mononuclear cells (PBMCs), and multiple cell subpopulations, such as B cells, CD4^+^ T cells, CD3^+^ cells, and monocytes in patients with SLE. The *PRDX6* protein expression level was also increased in patients with SLE compared with healthy donors.

**Conclusion:**

Our study provides new evidence that variants located in lncRNA coding genes are associated with SLE susceptibility.

## Introduction

Systemic lupus erythematosus (SLE; OMIM 152700) is a complex, multisystem autoimmune disease that is characterized by the production of autoantibodies with immune complex deposition leading to multiple organ damage (1, 2). The pathogenesis of SLE is still largely unknown, and it is believed that a combination of genetic and environmental factors contributes greatly to the etiology of SLE. Over recent years, increasing evidence has demonstrated that the dysregulation of long non-coding RNAs (lncRNAs) is involved in the pathogenesis of SLE (3, 4).

Long non-coding RNAs (lncRNAs) are a family of non-coding RNAs (ncRNAs) of more than 200 nucleotides in length that do not appear to code functional proteins (5). lncRNAs function both in *cis* and in *trans* to regulate gene expression and are involved in regulating various biological processes, such as cell proliferation, apoptosis, inflammation, and immune responses (6). Given the diverse cellular biological processes in which lncRNAs participate, lncRNAs have been implicated in a variety of diseases, including autoimmune diseases such as SLE.

The expression of lncRNAs is dysregulated in SLE and is correlated with clinical manifestations and organ impairments. The lncRNA expression profile in SLE was significantly changed, as there were 3,657 upregulated lncRNAs and 5,211 downregulated lncRNAs in SLE compared with the healthy group (7). Lnc-DC and growth-arrest-specific transcript 5 (GAS5) were downregulated in the plasma of patients with SLE, and their expression levels were negatively correlated with C3 expression and disease activity, respectively (8). nuclear paraspeckle assembly transcript 1 (NEAT1) expression was significantly upregulated in peripheral blood mononuclear cells (PBMCs) in patients with SLE and was positively correlated with disease activity (9). The expression of metastasis associated lung adenocarcinoma transcript 1 (MALAT1) was not only significantly higher in patients with SLE but was also revealed to be associated with interferon (IFN) signatures (10, 11).

In addition to detection results from clinical samples and experimental animal data, a growing body of evidence suggests that single nucleotide polymorphisms (SNPs) located in lncRNA coding regions are associated with SLE susceptibility. Located in an intronic enhancer, rs13259960 modulates SLEAR expression by impairing signal transducer and activator of transcription 1 (STAT1) recruitment and confers a predisposition to SLE (12). Variants rs205764 and rs547311 contributed to SLE susceptibility by enhancing linc00513 promoter activity, leading to the increased expression of linc00513 in SLE (13).

**Figure 1:**
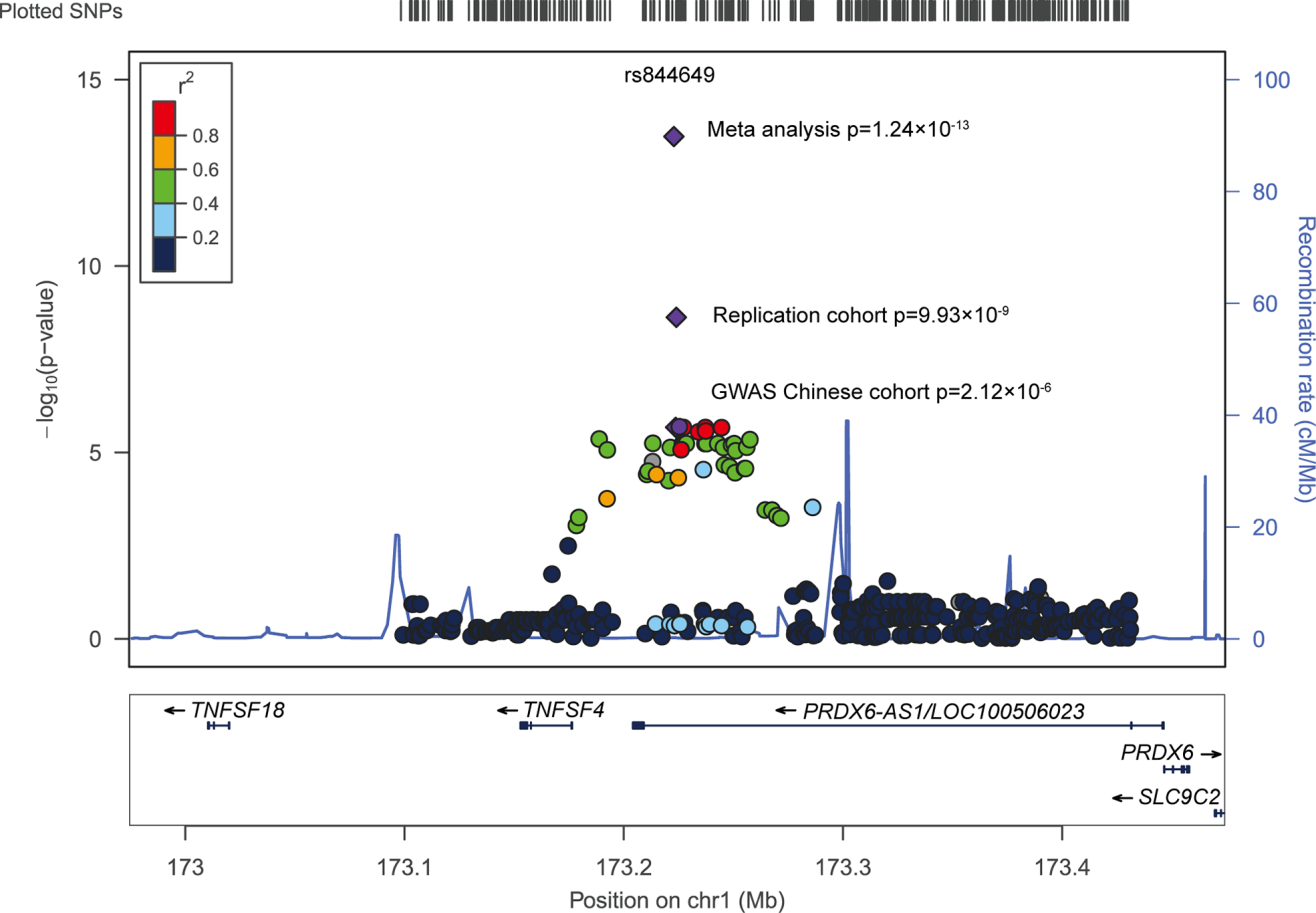
Regional association plots around rs844649 *PRDX6-AS1*.

A group of signals concentrated at *PRDX6-AS1* was identified when we performed an in-depth data analysis of previous genome-wide association studies (GWAS) (**Figure 1**). Although these groups of SNPs did not reach statistical significance in the Chinese population, we consider that the limited sample size (490 patients with SLE and 493 healthy donors) might be one of the most important reasons for this. Therefore, in our present study, we replicated the top signal in an independent cohort (1,003 patients with SLE and 815 healthy donors) from the Chinese population. We aimed to explore whether the genetic association signals at *PRDX6-AS1* were consistent and aimed to identify its possible function in the pathogenesis of SLE.

## Methods

### Study subjects

The GWAS Chinese cohort was recruited from Beijing, and it included 490 SLE patients and 493 healthy donors (14). The independent replication cohort, which was enrolled in Henan, consisted of 1,003 patients with SLE (age, 34.5 ± 12.8; female patients, 92.9%) and 815 geographically and ethnically matched healthy donors (age, 45.4 ± 15.7; female donors 49.8%). The individuals diagnosed with SLE fulfilled the revised diagnostic criteria of the American College of Rheumatology (ACR) (15). Written informed consent was obtained from all study subjects, and the study was approved by the Medical Ethics Committee of the First Affiliated Hospital of Zhengzhou University (2019-KY-247).

### SNP selection

*PRDX6-AS1* is located at chr1:173204199-173446294. It spans a region of approximately 240 kb, and 297 SNPs were covered by previous GWAS (14). Functional annotations were performed among genetically associated SNPs with *P*<0.05 to explore potential regulatory functions. The top signal was selected for replication.

### DNA extraction and genotyping

DNA extraction was performed with a DNA extraction kit (Qiagen, Hilden, Germany). The genotyping for the replication cohort was conducted by the Sequenom MassARRAY platform (Sequenom, Inc., San Diego, CA, USA), and the genotyping yield was higher than 99.5%.

### Differential expression analysis

Differential expression at the mRNA level was analyzed based on the ArrayExpress Archive database (http://www.ebi.ac.uk/arrayexpress) (16). It included immune cell subsets (B cells and CD4^+^ T cells E-GEOD-4588, CD3^+^ cells E-GEOD-13887, monocyte E-GEOD-46907) (17, 18), PBMCs (E-GEOD-50772) (19), and peripheral blood cells (E-GEOD-20864) (20). The protein level of *PRDX6* in sera was also analyzed using data derived from E-MTAB-5900.

### Statistical analysis

The distribution of the *PRDX6-AS1* rs844649 genotypes was tested for the Hardy–Weinberg equilibrium with the goodness-of-fit χ^2^ test, which did not violate the Hardy–Weinberg equilibrium (*P*=0.711 in healthy control individuals in the replication cohort). Genetic association was assessed by a two-tailed χ2 test, and the meta-analysis was used to test Cochran−Mantel−Haenszel statistics. Differential expression analysis was performed with Student’s t-test for continuous variables. Analyses were implemented using the SPSS 19.0 (SPSS, Chicago, IL, USA) and (SAS 9.3; SAS Institute,Cary, NC) software.

## Results

### *PRDX6-AS1* rs844649 polymorphisms associated with SLE susceptibility

A total of 43 SNPs were significantly associated with SLE susceptibility (*P*<0.05) in the Chinese population (**Supplementary Table 1**) (14). To reduce the probability of false-positive results caused by the number of tests, Bonferroni correction was applied for multi-testing correction. The level of significance was set at 1.68×10^-4^, as determined by dividing 0.05 by the number of tests. A total of 33 SNPs were associated with SLE susceptibility with *P*<1.68×10^-4^. The strongest association signal was detected at rs844649 (*P*=2.12×10^-6^, OR 1.55, 95% CI: 1.29–1.85) (**Figure 1** and **Table 1**).

**Table 1:** Association of rs844649 *PRDX6*-*AS1* with systemic lupus erythematosus (SLE) susceptibility.

Chr	Gene	SNP	Position (hg19)	Minor allele	GWAS Chinese cohort (490/493)			Replication cohort (1003/815)			Meta-analysis	
					MAF (case/control %)	*P*-value	OR (95% CI)	MAF (case/control %)	*P*-value	OR (95% CI)	*P*-value	OR (95% CI)
1	*PRDX6-AS1*	rs844649	173224343	C	47.1/36.6	2.12×10^-6^	1.55 (1.29–1.85)	47.9/38.5	9.93×10^-9^	1.47 (1.29–1.68)	1.24×10^-13^	1.50 (1.34–1.67)

Based on the results obtained from the above analysis, an independent cohort was enrolled to replicate the association signal at *PRDX6-AS1* rs844649. It was observed that the frequency of the risk allele C was 47.9% in patients with SLE and 38.5% in healthy donors, which was similar to the frequency of the GWAS Chinese cohort. The genotype frequencies of *PRDX6-AS1* rs844649 in patients and control individuals are presented in **Table 2**. Carriers of the rs844649 C allele were significantly associated with an increased risk of SLE after adjusting for age and sex.

**Table 2:** Genetic association analysis between rs844649 *PRDX6*-*AS1* with systemic lupus erythematosus (SLE) risk in replication cohort.

Genotype	Case (n=1003)	Control (n=815)	Crude OR (95% CI)	*P*-value	Adjusted OR (95% CI)^*^	*P*-value^*^
TT	272 (27.28)	310 (38.13)	1.000		1.000	
CT	495 (49.65)	380 (46.74)	1.489 (1.206–1.837)	2.03×10^-4^	1.291 (1.001–1.666)	4.96×10^-2^
CC	230 (23.07)	123 (15.13)	2.137 (1.627–2.807)	4.82×10^-8^	1.695 (1.220–2.353)	1.62×10^-3^
Additive			1.463 (1.280–1.673)	2.7×10^-8^	1.299 (1.106–1.526)	1.46×10^-3^
Dominant	725	503	1.643 (1.347–2.004)	9.6×10^-7^	1.391 (1.094–1.770)	7.26×10^-3^
Recessive	767	690	1.682 (1.321–2.142)	2.49×10^-5^	1.451 (1.085–1.941)	1.22×10^-2^

*Adjusted for age and gender.

Combining results from the GWAS Chinese cohort and our replication cohort, we pursued a meta-analysis approach and found a pronounced genetic association between *PRDX6-AS1* rs844649 and SLE susceptibility (p_meta_=1.24×10^-13^, OR 1.50, 95% CI: 1.34–1.67) (**Figure 1** and **Table 1**).

### Functional annotations

To explore the possible function of rs844649, RegulomeDB and HaploReg were applied to annotate the non-coding genome with known and predicted regulatory elements (21, 22). The RegulomeDB rank was 4, and the score was 0.60906 for rs844649. These findings indicate a potential regulatory function (**Table 3**). Data from HaploReg showed that rs844649 was located within the region of enhancer histone marks in six tissues, DNAse in five tissues, and in CTCF binding and motif change regions (Arnt, Dlx3, and Myc) (**Table 3**). Unfortunately, no expression quantitative trait loci (eQTL) effect was predicted to be associated with rs844649.

**Table 3:** Functional annotations by RegulomeDB and HaploReg.

SNPs	RegulomeDB		HaploReg							
	Rank	Score	Promoter histone marks	Enhancer histone marks	DNAse	Proteins bound	Motifs changed	NHGRI/EBI GWAS hits	GRASP QTL hits	Selected eQTL hits
rs844644	5	0.13454	–	–	–	–	Pou5f1, ZEB1	–	1	–
rs844645	4	0.60906	–	–	–	–	4 altered motifs	–	–	–
rs74448919	5	0	–	–	–	–	3 altered motifs	–	–	1
rs12039904	5	0.13454	–	–	–	–	Esr2, THAP1, YY1	–	–	–
rs2795288	5	0.13454	–	–	–	–	Pou2f2, Pou3f2	–	–	1
rs1012507	5	0.14776	–	–	–	–	6 altered motifs	–	1	3
rs35086785	5	0.13454	–	–	–	–	CEBPB	–	–	–
rs844648	2b	0.66261	–	BLD, MUS	–	–	14 altered motifs	–	1	1
rs844649	4	0.60906	–	6 tissues	5 tissues	CTCF	Arnt, Dlx3, Myc	–	–	–
rs844651	5	0.13454	–	MUS, BLD	–	–	Evi-1, GATA, TAL1	–	–	–
rs12048385	4	0.60906	–	HRT, MUS, BLD	–	–	–	–	–	–
rs704840	4	0.60906	–	MUS, BLD	BLD	–	Evi-1	1	1	–
rs2840317	5	0.13454	–	MUS, BLD	MUS	–	BATF, GR	–	1	–
rs2901716	4	0.60906	–	8 tissues	MUS	–	GATA, SP1	–	–	–
rs844655	7	0.18412	–	BLD	–	–	Nkx2, Rad21	–	–	–
rs10912573	7	0.18412	–	–	–	–	–	–	–	–
rs10489265	7	0.18412	–	–	–	–	4 altered motifs	–	1	–
rs844659	4	0.60906	–	9 tissues	4 tissues	–	GATA, TAL1	–	–	–
rs844660	4	0.60906	–	9 tissues	CRVX, BRN, SKIN	–	–	–	–	–
rs34313362	4	0.60906	ESDR	9 tissues	14 tissues	BCL11A, EBF1, P300	Maf, RFX5	–	–	–
rs12046550	5	0.03648	–	OVRY, MUS	ADRL	–	HNF4, PPAR, RXRA	–	–	–
rs844663	5	0.30371	–	ESDR	–	–	5 altered motifs	–	–	–
rs12403570	4	0.60906	ESDR	6 tissues	SKIN	–	DBP	–	–	–
rs10912577	4	0.60906	–	–	IPSC	–	9 altered motifs	–	–	–
rs12049190	6	0.11093	–	–	–	–	7 altered motifs	–	–	3
rs35634597	7	0.18412	–	ESC, IPSC	–	–	–	–	–	–
rs67638449	7	0.18412	–	ESDR, ESC, IPSC	–	–	–	–	–	–
rs12750070	6	0.39217	–	ESDR, ESC	–	–	GCM	–	–	2
rs12405577	5	0.13454	–	ESDR, ESC, MUS	–	–	Hoxa3, Hoxa4, Mef2	–	–	–
rs6697570	5	0.34168	–	–	–	–	5 altered motifs	–	–	3
rs12143114	4	0.60906	–	–	–	MEF2A, MEF2C	Mef2	–	–	–
rs35691278	6	0	–	–	–	–	4 altered motifs	–	–	–
rs10912580	6	0.59	–	–	–	–	5 altered motifs	–	–	–
rs10798266	5	0.58955	–	–	–	FOXA1	Brachyury	–	–	–
rs4916319	4	0.60906	BLD	GI	–	–	Nkx3, Pax-5, SP2	–	–	–
rs4916213	6	0.4855	–	BLD	–	–	SRF	–	–	–
rs1342032	3a	0.42363	–	5 tissues	CRVX	4 bound proteins	–	–	2	–
rs73037142	7	0.18412	–	–	–	–	Hltf	–	–	–
rs1539261	5	0.005	–	4 tissues	SKIN, ADRL	–	Hsf	–	–	–
rs7526970	2b	0.58729	BLD	12 tissues	11 tissues	5 bound proteins	8 altered motifs	–	–	–
rs16845703	5	0.13454	–	SKIN	SKIN	–	PU.1	–	–	–
rs4279882	3a	0.57565	BLD	8 tissues	4 tissues	–	9 altered motifs	–	–	1
rs6425230	3a	0.61268	BLD	6 tissues	–	–	Ik-1, RXRA, ZBTB7A	–	–	–

BLD, blood; MUS, muscle; HRT, heart; OVRY, ovary; ESDR, ES-deriv; ESC, embryonic stem cell; IPSC, induced pluripotent stem cell; CRVX, cervix; BRN, brain; SKIN, skin; ADRL, adrenal; GI, digestive.

We further expanded our annotation to all 43 genetically associated SNPs with *P*<0.05. The rs844648 and rs7526970 variants had a RegulomeDB rank of 2b (ranks 1 to 7 represent decreasing regulatory evidence), and rs844648 had the highest RegulomeDB score of 0.66261 (**Table 3**).

### Expression analysis of *PRDX6*

**Figure 2:**
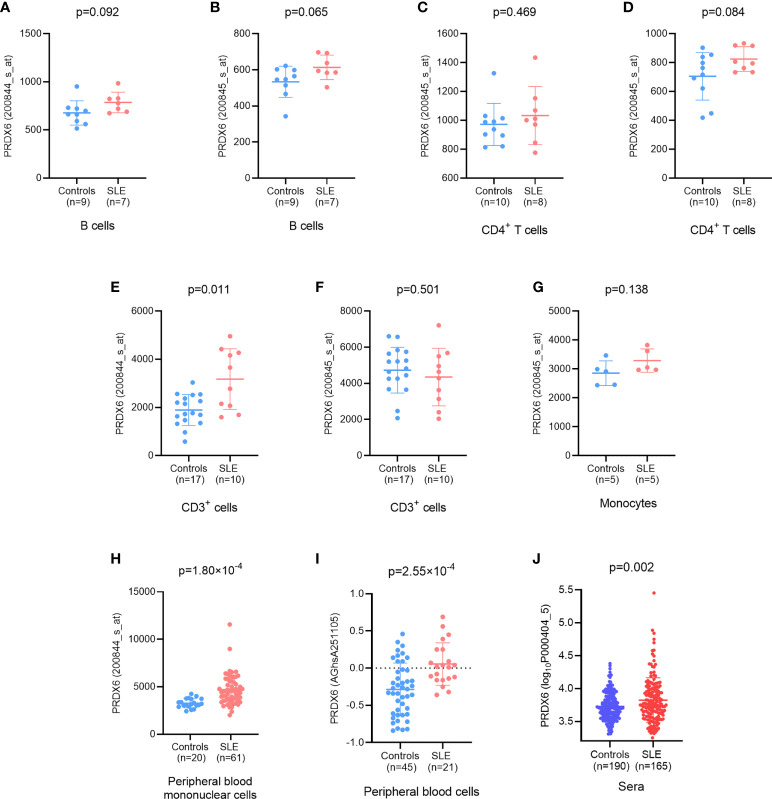
Differential expression of PRDX6 between patients with systemic lupus erythematosus (SLE) and controls. **(A, B)** At the mRNA level, the expression of PRDX6 was elevated in B cells (E-GEOD-4588); **(C, D)** CD4^+^ T cells (E-GEOD-4588); **(E, F)** CD3^+^ cells (E-GEOD-13887); **(G)** monocytes (E-GEOD-46907); **(H)** Peripheral blood mononuclear cells (PBMCs) (E-GEOD-50772); and **(I)** peripheral blood cells. **(J)** The level of *PRDX6* was also significantly elevated in patients with SLE compared with healthy donors at protein level (E-MTAB-5900). The analyses were performed based on information available in online databases as referenced.

The expression of *PRDX6* was elevated in B cells (**Figures 2A, B**) and CD4^+^ T cells (**Figures 2C, D**) in patients with SLE compared with healthy control individuals and approached marginal statistical significance. In CD3^+^ cells, the expression of *PRDX6* was significantly increased in patients with SLE compared with healthy donors (detected by 200844_s_at, *P*=0.001) (17) (**Figures 2E, F**). The expression of *PRDX6* demonstrated an increasing trend in monocytes in patients with SLE, but this was without statistical significance. This might be due to the limited sample size (18) (**Figure 2G**).

For PBMCs, in comparison to that in healthy subjects, the level of *PRDX6* was significantly upregulated in patients with SLE (*P*=1.8×10^-4^) (19) (**Figure 2H**). A markedly increased level of *PRDX6* expression was also identified in peripheral blood cells from patients with SLE (*P*=2.55×10^-4^) (20) (**Figure 2I**).

The analysis above was performed at the mRNA level, and the *PRDX6* protein expression level was determined using data from E-MTAB-5900 hereafter. According to the data from the protein microarray, the level of *PRDX6* was significantly elevated in patients with SLE compared with healthy donors (*P*=0.002) (**Figure 2J**).

## Discussion

Although accumulated evidence suggests that the dysregulation of lncRNAs is involved in the pathogenesis of SLE, the genetic contributions of lncRNA coding genes to SLE susceptibility remain largely unknown. In our present study, we identified that the rs844649 *PRDX6-AS1* variant located on chromosome 1q25.1 was associated with SLE susceptibility, reaching genome-wide significance in two independent cohorts from China. The 1q25.1 region is a hot spot containing potential SLE susceptibility loci. It is well established that tumor necrosis factor ligand superfamily member 4 (TNFSF4) gene polymorphisms are associated with SLE susceptibility in large sample sizes and diverse multiracial and multiethnic populations (23–30). A previous study also revealed that rs10798269, located on 1q25.1, was associated with SLE in women by a genome-wide association scan (31). Despite our data for rs844649 *PRDX6-AS1* being from a Chinese population, genetic association results from a Korean population (1,710 patients with SLE vs. 3,167 control individuals) showed a more remarkable result (*P*=1.67 × 10^-12^, OR 1.37, 95% CI: 1.25–1.49) (14). However, additional studies are still needed to validate the signal from rs844649 *PRDX6-AS1* on chromosome 1q25.1 in more centers with different ethnicities from different centers.

To explore the possible regulatory function of rs844649, we performed functional annotations using a publicly available database. It was observed that rs844649 was located within the regions of enhancer histone marks, DNAse, CTCF binding, and motif changes, but no eQTL effect was identified. Thus, future studies are needed to search for the causal SNP on *PRDX6-AS1* from a functional perspective.

Genetic association analysis indicated that the lncRNA *PRDX6-AS1* might be involved in the pathogenesis of SLE. Given that one of the first critical functions of a certain lncRNA is to regulate the expression of adjacent protein coding genes, we further examined the expression level of *PRDX6* based on an array expression database. Importantly, the mRNA expression of *PRDX6* was elevated in peripheral blood cells, PBMCs, and multiple cell subpopulations, including B cells, CD4^+^ T cells, CD3^+^ cells, and monocytes, in patients with SLE. The *PRDX6* protein expression level was also increased in patients with SLE compared with healthy donors.

Peroxiredoxin (*PRDX*) 6 belongs to the *PRDX* family, and three enzymatic activities have been identified for it. These activities are peroxidase, phospholipase A2 (PLA2), and acyl transferase activities (32). Recent advances have highlighted the protective role of *PRDX6* against the pathogenesis of SLE through studies using *cis*-eQTL analysis and *Prdx6* knockout (KO) mice (33). SLE risk-associated SNPs downregulate the expression of *PRDX6*, and *Prdx6* deficiency upregulates antibody production (33, 34). Our study revealed that the expression of *PRDX6* was upregulated in patients with SLE, as demonstrated by the integrated analysis of multiple studies in different cell subtypes. The increased expression of *Prdx6* has been demonstrated in various injury models and patients with peripheral arterial disease (35–37). Whether *Prdx6* has protective antioxidant functions or participates in redox imbalance by redox signaling remains controversial. Considering the dual role of *PRDX6* in redox imbalance in inflammation models, whether the elevated level of *PRDX6* was simply a biomarker of SLE or a driver of disease pathogenesis warrants further investigation.

## Conclusion

In this study, we performed a genetic association analysis among a Chinese population and confirmed that the rs844649 *PRDX6-AS1* variant on chromosome 1q25.1 was significantly associated with SLE susceptibility. Moreover, *PRDX6*, the closest gene to *PRDX6-AS1*, was upregulated in patients with SLE in multiple cell subpopulations. Our study provides new evidence that variants located in lncRNA coding genes are associated with SLE susceptibility.

## Data availability statement

The data sets presented in this study can be found in online repositories. The names of the repository/repositories and accession numbers can be found in the article/**Supplementary Material**.

## Ethics statement

The study was approved by the Medical Ethics Committee of the First Affiliated Hospital of Zhengzhou University (2019-KY-247). The patients/participants provided their written informed consent to participate in this study.

## Author contributions

Y-YQ and Z-ZZ conceived and designed the experiment. Y-YQ, X-XZ, and X-RL performed the experiments. X-XZ, J-PY, Y-FZ, and YC analyzed the data. Y-YQ, X-XZ, J-PY, and Z-ZZ interpreted the findings. All the authors contributed to writing the article. All authors contributed to the article and approved the submitted version.

## Funding

This work was supported by the National Natural Science Foundation of China [grant number 81900643, 81873611]; the China Postdoctoral Science Foundation [grant number 2019M652592]; the funders had no role in study design, data collection, and analysis, decision to publish, or preparation of the manuscript.

## Acknowledgments

We thank all the members of the laboratory for their technical assistance. We also thank the patients and their families, and the healthy donors for their cooperation and for giving consent to participate in this study.

## Conflict of interest

The authors declare that the research was conducted in the absence of any commercial or financial relationships that could be construed as a potential conflict of interest.

## Publisher’s note

All claims expressed in this article are solely those of the authors and do not necessarily represent those of their affiliated organizations, or those of the publisher, the editors and the reviewers. Any product that may be evaluated in this article, or claim that may be made by its manufacturer, is not guaranteed or endorsed by the publisher.

## Abbreviations

SLE, systemic lupus erythematosus; GWAS, genome-wide association study; SNP, single nucleotide polymorphism; lncRNA, long non-coding RNAs; *PRDX6*, peroxiredoxin 6; PBMCs, peripheral blood mononuclear cells.
