# Supplementary material for: PRDX6AS1 gene polymorphisms and SLE susceptibility in Chinese populations
Source: Front Immunol. 2022 Oct 12;13:987385. doi: 10.3389/fimmu.2022.987385 (PMC9601311; doi:10.3389/fimmu.2022.987385)
Supplement: Supplementary file 1 [file Table_1.docx]

***PRDX6-AS1* gene polymorphisms and SLE susceptibility in Chinese populations**

Xiao-Xue Zhang^1#^, Jun-peng You^1#^, Xin-Ran Liu^1^, Ya-Fei Zhao^1^, Yan Cui^12^, Zhan-Zheng Zhao^1*^, Yuan-Yuan Qi^1*^

**AUTHORS’ INSTITUTION AND AFFILIATION**

1. Nephrology Hospital, the First Affiliated Hospital of Zhengzhou University, Zhengzhou University, Henan 4500052, China;
2. Department of Nephrology, Wuhan Fourth Hospital, Puai Hospital, Tongji Medical College, Huazhong University of Science and Technology, Wuhan, Hubei, China.

CORRESPONDING AUTHOR

Dr. Yuan-yuan Qi, MD & PhD;

Email: qqyyiillyy@126.com

Nephrology Hospital, the First Affiliated Hospital of Zhengzhou University,

Institute of Nephrology, Zhengzhou University

No.1, Jianshe Road, Erqi District

Dr. Zhan-zheng Zhao, MD & PhD;

Email: zhanzhengzhao@zzu.edu.cn

Nephrology Hospital, the First Affiliated Hospital of Zhengzhou University,

Institute of Nephrology, Zhengzhou University

No.1, Jianshe Road, Erqi District

Zhengzhou 4500052, P.R China

Supplementary table1. Association results of SNPs in PRDX6-AS1 and SLE susceptibility (Sun et al., 2016).

| Chr. | SNPs | Pos (hg19) | Minor allele | MAF (Case/Control, %) | P-value | OR(95%CI) |
| --- | --- | --- | --- | --- | --- | --- |
| 1 | rs844644 | 173209495 | C | 50.9/41.7 | 4.03×10^-5^ | 1.45(1.22-1.73) |
| 1 | rs844645 | 173210090 | G | 50.9/41.6 | 3.3×10^-5^ | 1.46(1.22-1.74) |
| 1 | rs74448919 | 173212143 | C | 51.7/42.1 | 1.83×10^-5^ | 1.48(1.23-1.76) |
| 1 | rs12039904 | 173212273 | T | 34.4/25.1 | 5.9×10^-6^ | 1.57(1.29-1.91) |
| 1 | rs2795288 | 173213935 | A | 51.7/42.5 | 4.07×10^-5^ | 1.45(1.21-1.73) |
| 1 | rs1012507 | 173219471 | T | 36/27.6 | 5.93×10^-5^ | 1.48(1.22-1.79) |
| 1 | rs35086785 | 173220249 | G | 34.5/25.3 | 7.66×10^-6^ | 1.56(1.28-1.89) |
| 1 | rs844648 | 173223863 | A | 51.7/42.6 | 4.94×10^-5^ | 1.44(1.21-1.73) |
| 1 | rs844649 | 173224343 | C | 47.1/36.6 | 2.12×10^-6^ | 1.55(1.29-1.85) |
| 1 | rs844651 | 173225144 | G | 48/38 | 8.77×10^-6^ | 1.5(1.25-1.80) |
| 1 | rs12048385 | 173225759 | T | 34.5/25.2 | 6.02×10^-6^ | 1.57(1.29-1.90) |
| 1 | rs704840 | 173226195 | G | 47.1/36.6 | 2.22×10^-6^ | 1.54(1.29-1.85) |
| 1 | rs2840317 | 173226498 | A | 34.5/25.2 | 6.02×10^-6^ | 1.57(1.29-1.90) |
| 1 | rs2901716 | 173227515 | A | 34.5/25.2 | 6.02×10^-6^ | 1.57(1.29-1.90) |
| 1 | rs844655 | 173233114 | C | 47/36.6 | 2.91×10^-6^ | 1.54(1.28-1.84) |
| 1 | rs10912573 | 173235319 | T | 35.3/26.6 | 3×10^-5^ | 1.51(1.24-1.83) |
| 1 | rs10489265 | 173236065 | C | 34.5/25.2 | 6.02×10^-6^ | 1.57(1.29-1.90) |
| 1 | rs844659 | 173236216 | T | 47.1/36.6 | 2.22×10^-6^ | 1.54(1.29-1.85) |
| 1 | rs844660 | 173236355 | G | 47/36.6 | 2.77×10^-6^ | 1.54(1.28-1.84) |
| 1 | rs34313362 | 173236716 | C | 34.5/25.2 | 6.02×10^-6^ | 1.57(1.29-1.90) |
| 1 | rs12046550 | 173241872 | A | 34.5/25.2 | 6.02×10^-6^ | 1.57(1.29-1.90) |
| 1 | rs844663 | 173243581 | C | 47.2/36.7 | 2.24×10^-6^ | 1.54(1.29-1.85) |
| 1 | rs12403570 | 173244448 | T | 34.5/25.3 | 7.66×10^-6^ | 1.56(1.28-1.89) |
| 1 | rs10912577 | 173244862 | T | 35.3/26.5 | 2.24×10^-5^ | 1.52(1.25-1.84) |
| 1 | rs12049190 | 173247257 | A | 35.3/26.5 | 2.54×10^-5^ | 1.51(1.25-1.83) |
| 1 | rs35634597 | 173248183 | T | 34.5/25.2 | 6.51×10^-6^ | 1.56(1.29-1.90) |
| 1 | rs67638449 | 173249338 | T | 34.4/25.1 | 6.05×10^-6^ | 1.57(1.29-1.91) |
| 1 | rs12750070 | 173249772 | T | 35.1/26.5 | 3.63×10^-5^ | 1.5(1.24-1.82) |
| 1 | rs12405577 | 173249994 | T | 34.3/25.2 | 9.37×10^-6^ | 1.55(1.28-1.89) |
| 1 | rs6697570 | 173254103 | C | 35.3/26.6 | 2.8×10^-5^ | 1.51(1.24-1.83) |
| 1 | rs12143114 | 173254508 | C | 35.3/26.6 | 2.8×10^-5^ | 1.51(1.24-1.83) |
| 1 | rs35691278 | 173255118 | T | 34.4/25.2 | 7.52×10^-6^ | 1.56(1.28-1.90) |
| 1 | rs10912580 | 173256550 | G | 34.5/25.1 | 4.71×10^-6^ | 1.58(1.30-1.92) |
| 1 | rs10798266 | 173263524 | G | 43.8/35.9 | 3.63×10^-4^ | 1.39(1.16-1.67) |
| 1 | rs4916319 | 173266578 | G | 43.9/36 | 3.65×10^-4^ | 1.39(1.16-1.67) |
| 1 | rs4916213 | 173268821 | T | 43.7/36 | 5.14×10^-4^ | 1.38(1.15-1.65) |
| 1 | rs1342032 | 173270649 | A | 43.3/35.7 | 6×10^-4^ | 1.37(1.15-1.65) |
| 1 | rs73037142 | 173282296 | T | 13/16.2 | 4.85×10^-2^ | 0.78(0.60-1.00) |
| 1 | rs1539261 | 173285148 | C | 30.1/22.9 | 3.09×10^-4^ | 1.45(1.18-1.77) |
| 1 | rs7526970 | 173299064 | A | 47.4/42.7 | 3.43×10^-2^ | 1.21(1.01-1.45) |
| 1 | rs16845703 | 173319294 | G | 17.2/13.7 | 2.94×10^-2^ | 1.31(1.03-1.68) |
| 1 | rs4279882 | 173387942 | C | 1.5/2.8 | 4.72×10^-2^ | 0.53(0.28-1.00) |
| 1 | rs6425230 | 173388038 | A | 1.4/2.7 | 4.22×10^-2^ | 0.51(0.27-0.99) |

Sun, C., Molineros, J.E., Looger, L.L., Zhou, X.J., Kim, K., Okada, Y., Ma, J., Qi, Y.Y., Kim-Howard, X., Motghare, P., Bhattarai, K., Adler, A., Bang, S.Y., Lee, H.S., Kim, T.H., Kang, Y.M., Suh, C.H., Chung, W.T., Park, Y.B., Choe, J.Y., Shim, S.C., Kochi, Y., Suzuki, A., Kubo, M., Sumida, T., Yamamoto, K., Lee, S.S., Kim, Y.J., Han, B.G., Dozmorov, M., Kaufman, K.M., Wren, J.D., Harley, J.B., Shen, N., Chua, K.H., Zhang, H., Bae, S.C., and Nath, S.K. (2016). High-density genotyping of immune-related loci identifies new SLE risk variants in individuals with Asian ancestry. *Nat Genet* 48**,** 323-330.
